# Comparison between Two Generic Questionnaires to Assess Satisfaction with Medication in Chronic Diseases

**DOI:** 10.1371/journal.pone.0056247

**Published:** 2013-02-20

**Authors:** Stéphanie Delestras, Matthieu Roustit, Pierrick Bedouch, Mélanie Minoves, Valérie Dobremez, Roseline Mazet, Audrey Lehmann, Magalie Baudrant, Benoît Allenet

**Affiliations:** 1 Department of Pharmacy, Grenoble University Hospital, Grenoble, France; 2 Clinical Research Centre, Inserm CIC003, Grenoble University Hospital, Grenoble, France; 3 TIMC UMR CNRS 5525, Grenoble, France; 4 Joseph Fourier University, Grenoble, France; University Hospital La Paz, Spain

## Abstract

**Objective:**

The objective of this work was to compare two generic questionnaires assessing patients’ satisfaction with medication. In addition we tested whether satisfaction can predict adherence to medication regimens in patients with chronic diseases, and which dimensions of satisfaction are most involved.

**Methods:**

This prospective, observational study was conducted over one year in a heterogeneous population of patients with various chronic diseases. Satisfaction with medication was assessed by using the TSQM® vII and the SatMed-Q® questionnaires, and adherence to treatment was assessed with the Morisky-Green questionnaire. Clinical pharmacists interviewed patients to collect clinical, demographic and therapeutic data.

**Results:**

190 patients were enrolled. Both questionnaires showed excellent reliability and correlation was high (R = 0.70; p<0.001). Adherence was correlated with satisfaction with medication whether assessed with the SatMed-Q® (R = 0.23; p = 0.002) or the TSQM® (R = 0.17; p = 0.02). Among different dimensions of satisfaction, convenience of use and side effects are prominent predictors of adherence.

**Conclusion:**

Adherence is related to the patient’s satisfaction with medication whether assessed with the TSQM® vII or the SatMed-Q®. Therefore, these simple questionnaires could be used as predictive tools to identify patients whos’ adherence needs to be improved.

## Introduction

Sub-optimal adherence to medication leads to a loss of opportunities in clinical terms, affects quality of life and has economic consequences [1], [2]. Previous surveys of adherence give highly heterogeneous results, are difficult to interpret because of differences in terminology and definitions between studies [3] and use a variety of methods of assessment [4]. They are usually based on drug dispensary records expressed as “Medication Possession Ratio”, and self reports [5]. They also differ in the profile of the patients included (heterogeneity in terms of severity of disease and management strategy).

Satisfaction with medication can be defined as the patient’s evaluation of the process of taking the medication, its short term effects and the longer term outcomes associated with it. An association between adherence and treatment satisfaction has been recently observed [6]. Indeed, satisfaction with treatment associated with a positive experience can induce motivation to adhere in the long term, and dissatisfaction with some aspect can easily jeopardize the patient’s final willingness to continue taking the medication. Therefore, satisfaction could be a useful indicator of adherence.

Satisfaction has been used as an outcome in many clinical trials involving a wide variety of conditions such as type 2 diabetes, schizophrenia, or migraine [7], [8], [9], [10], [11], in certain situations: when different routes of administration, dose regimens or side effect profiles have been compared for drugs of similar efficacy; when demonstration of satisfaction with a medication relative to a comparator is considered to offer a potential advantage with respect to adherence issues.

Patients’ satisfaction with medication has been shown to be related to adherence in chronic diseases [12], [13], [14], [15], [16]. Due to the prolonged and progressive nature of many chronic diseases poor adherence, which often begins after several months of treatment, can adversely affect the long term effectiveness of a drug [17]. Thus, an assessment of the patient’s satisfaction could be useful to help identify those at risk of poor adherence, and enable clinicians to target their interventions toward the aspects responsible for this.

Most studies of satisfaction with medication have used disease-specific tools. However, for widespread use in routine daily practice, generic questionnaires would be more suitable. To date, to our knowledge, just two generic questionnaires, suitable for use in any chronic disease, have been developed to assess patient’s satisfaction. The Treatment Satisfaction Questionnaire for Medication (TSQM®) [18] and its abridged versions (TSQM® vII [19] and more recently TSQM-9® [20]) explore four dimensions: effectiveness, side effects, ease and comfort of use, and the patient’s general opinion regarding the treatment. The second is the Treatment Satisfaction With Medicines Questionnaire (SatMed-Q®) [21]. This explores the same dimensions as the TSQM® and two additional dimensions (impact of the treatment on daily life and quality of monitoring by health professionals). These extra dimensions may provide an advantage of SatMed-Q® over TSQM®. However, to our knowledge, this has never been tested. Then, although satisfaction has been shown to be correlated with adherence for each of these tools, it seems interesting to test whether one of them appears to be a better predictor of patient’s adherence than the other.

Moreover, the impact of the different dimensions of these questionnaires on adherence has never been tested. Identification of the dimensions that influence adherence could be helpful when personalizing patient information and education.

Finally, as quality of life assessment is widely used in clinical practice, it is interesting to measure the degree of correlation between quality of life and patient’s satisfaction with medication, and to investigate the influence of the different dimensions of patient’s satisfaction on mental and physical components of quality of life.

The primary objective of this work was to compare the two questionnaires by assessing the correlation between satisfaction and adherence to medication in a heterogeneous population of patients with various chronic diseases. As secondary objectives, we assessed which dimensions of satisfaction with medication could predict adherence, the correlation between patient satisfaction with medication and quality of life, and the influence of several demographic and therapeutic parameters on patients’ satisfaction with medication.

## Methods

### Ethics Statement

All patients were informed of the study before inclusion and ethical approval was obtained on April 2^nd^, 2010 (CECIC Rhône-Alpes-Auvergne, Clermont-Ferrand, Institutional Review Board 5891).

### Study Population

We included inpatients and outpatients from different departments of a large university hospital. They were aged 18 years or over, under stable drug therapy for at least 2 months at the time of inclusion, treated for one of the following chronic diseases: asthma or chronic obstructive pulmonary disease (COPD), type 2 diabetes, hepatitis B or C, pulmonary hypertension, Crohn's disease or ulcerative colitis (inflammatory bowel disease; IBD), cystic fibrosis (CF), rheumatoid arthritis (RA), ankylosing spondylitis (AS), acquired immunodeficiency syndrome (AIDS), or solid organ transplant recipients (SOTR). Interviews were conducted on predefined days. Randomization lists with room numbers were made for inpatients inclusion. At the beginning of each study day, interviews were planned according to the list, excluding patients already included on a previous day. For outpatients (day care and regular consultation) all eligible patients on the study day were proposed the study. Non-inclusion criteria included patients under judicial protection, as well as patients who did not speak French. To avoid under-representation, a disease group was not included in the analysis when the group had accrued 5 patients or less.

### Study Design and Data Collection

Included patients were interviewed by clinical pharmacists (SD, PB, MM, VD, RM, AL, MB, BA). After explaining the study, investigators collected data using a standardized form. The following data were collected: patient data (age, gender, level of education, marital status), disease data (disease, time since diagnosis) and drug therapy data (patient requiring assistance to manage medication or not, number of drugs prescribed for the chronic disease, time since the treatment was started, concomitant drugs, route of drug administration). At the end of the 30 min interview four self-administered questionnaires were left for the patient to complete alone and collected 30 min later.

### Questionnaires

Satisfaction with medication was assessed with the SatMed-Q® [21] and TSQM® vII [19] questionnaires. The SatMed-Q® contains 17 items, each scored on a 5-point Likert scale. The total composite score ranges between 0 and 68. The score was converted to a percentage as recommended by the author of the original version [21]. The TSQM® vII contains 11 items scored on a 5 or 7-point Likert scale [19]. Overall satisfaction with medication was assessed using the composite score of the SatMed-Q® and the ‘Global satisfaction’ dimension of the TSQM®. Clarity and ease of answering the questionnaires was rated on a 5-point Likert scale.

Patient’s adherence was measured by a self-administered questionnaire derived from the Morisky-Green Medication Adherence Questionnaire validated in French [22]. It includes six questions with Yes/No answers (each “Yes” scored as 1 point, with the final score ranging between 0 and 6). Adherence was rated as “good” (score = 0), “minor adherence problems” (score = 1 or 2) and “poor” (score>2) [22].

Quality of life was assessed using the SF-36® (Short Form Health Survey Questionnaire). This questionnaire assesses the physical and mental health of an individual with 36 questions about eight aspects of health (physical activity, physical ability to accomplish everyday tasks, physical pain, general health, vitality, social functioning, emotional state, perceived general mental health status). Scores for the physical (PCS) and mental s (MCS) domains were calculated.

### Linguistic Validation of the SatMed-Q®

The SatMed-Q® was originally developed and validated in Spanish. Between September and December 2009, before starting the present study, we performed a linguistic validation of the French SatMed-Q® in collaboration with the MAPI Research Institute (Lyon, France). Linguistic validation aims at providing translations that are conceptually equivalent to the original version, thus preserving the psychometric properties of the original tool. The linguistic validation was achieved through the following steps: 1. forward translation by two qualified translators; 2. reconciliation; 3. backward translation; 4. clinician’s review; 5. cognitive debriefing with 5 patients (suffering from asthma, heart failure, type 2 diabetes, pulmonary hypertension and RA) and 6. international harmonization with the author of the original version; this standardized methodology is described in details in the following reference [23]).

### Sample Size Calculation and Statistical Analysis

The authors of the original version of the SatMed-Q® have shown a correlation with the Morisky-Green questionnaire of 0.22 (Pearson correlation test). Based on comparable results, with the significance level α set at 0.05 and 80% power, 160 subjects were needed (nQuery Advisor ® 7.0, Statistical Solutions ltd., Cork, Ireland). Assuming a 5% rate of incomplete and therefore unusable questionnaires (the authors of the original reported a rate of 3.3%), we estimated that 170 patients should be included in our study.

Categorical data were reported as frequency and percentage and continuous data as mean and standard deviation. Internal consistency was evaluated with Cronbach’s alpha coefficient and the Pearson correlation coefficient was used for correlations between scores. The influence of the various dimensions of satisfaction with adherence was tested using multiple linear regression analysis. Between-group comparisons were analyzed by one-way ANOVA. *Post hoc* comparisons were performed using the Tukey test, or the Gabriel test if uneven numbers. When application conditions were not met (Levene's test was used to test the homogeneity of variance), nonparametric tests were used. We considered p-values <0.05 as significant, corrected by Bonferroni’s method for multiple comparisons. Statistical analysis was performed with SPSS 13.0 for Windows (SPSS® Inc, Chicago IL, USA).

## Results

### 1. Study Population

Among the 190 patients initially recruited, patients with CF (n = 2) were excluded due to under representation. Fourteen patients were also excluded because of missing data regarding the primary endpoint. Therefore, 172 patients were included in the analysis. The characteristics of the population are shown in Table 1. Mean adherence was 4.86±0.96, and quality of life was 43.38±11.2 and 40.87±10.6 for the mental and physical domains of the SF-36®, respectively.

**Table 1:** Characteristics of the population (N = 172).

**Age** (year)		51.5 (15.3)
**Sex**, male		86 (50.3)
**Level of education completed**		
	Primary school	25 (14.6)
	Middle school	34 (19.9)
	High school	47 (27.5)
	Undergraduate degree and higher	65 (38.0)
	MD	1 (1.7)
**Marital status**		
	Single	100 (58.5)
	Married, civil partnership, cohabiting	36 (21.1)
	Separated, divorced	24 (14.0)
	Widowed	11 (6.4)
	MD	1 (1.7)
**Situation at home**		
	Living alone	42 (24.4)
	Not living alone	130 (75.6)
**Chronic disease**		
	Asthma/Chronic obstructive pulmonary disease	11 (6.4)
	Type 2 Diabetes	31 (18.0)
	Hepatitis B or C	6 (3.5)
	Pulmonary hypertension	6 (3.5)
	Crohn's disease/Ulcerative colitis	32 (18.6)
	Rheumatoid arthritis	44 (25.6)
	Ankylosing spondylitis	16 (9.3)
	Acquired immunodeficiency syndrome	10 (5.8)
	Solid organ transplant	16 (9.3)
**Type of care**		
	Full hospitalization	58 (33.7)
	Day care	101 (58.7)
	Regular consultations	13 (7.6)
**Time since diagnosis**		
	<6 months	8 (4.7)
	6 months–1 year	8 (4.7)
	1–5 years	33 (19.2)
	6–10 years	39 (22.7)
	>10 years	84 (48.8)
**Date of initiation of current treatment**		
	2–6 months	33 (19.2)
	6 months–1 year	24 (14.0)
	1–5 years	77 (44.8)
	6–10 years	19 (11.0)
	>10 years	19 (11.0)
**Medication management**		
	Autonomous	144 (83.7)
	Requiring assistance	28 (16.3)
**Route of drug administration**		
	Only oral	56 (32.6)
	At least one injected drug	116 (67.4)

Age is expressed as mean (standard deviation). All other variables are expressed as numbers (percentage). MD: Missing Data.

### Satisfaction with Medication

Reliability was tested for each dimension of the two questionnaires. Cronbach’s alpha coefficients were around 0.9 for each dimensions of the two questionnaires, suggesting excellent internal consistency (Table 2).

**Table 2:** Reliability of each dimension of the satisfaction with medication questionnaires.

	Cronbach’s alpha coefficients	
	SatMed-Q®	TSQM® vII
Side effects	0.93	0.90
Effectiveness	0.87	0.86
Convenience of use	0.89	0.88
Overall satisfaction	0.88	0.88
Impact on daily activities	0.88	NA
Medical care	0.89	NA

Scores of satisfaction with medication for both questionnaires are reported in Table 3. Convergent validity was confirmed by the significant correlation between the two tools for each dimension and for global satisfaction (Table 3). Among the 150 subjects who rated both questionnaires for clarity and ease of answering, 57 favored the SatMed-Q® and 8 favored the TSQM® vII, while 85 patients did not express any preference. Mean scores on the 5-point Likert scale were 3.16±0.99 for the SatMed-Q® and 2.65±1.2 for the TSQM® vII (p<0.001, Wilcoxon rank test).

**Table 3:** Correlation between scores of satisfaction for each dimension of the questionnaires.

Dimensions	SatMed-Q®(of 12)	TSQM® vII(of 100)	R (p-value)
Effectiveness	8.53 (3.49)	61.62 (24.16)	0.26 (<0.001)
Side effects	9.49 (3.41)	77.06 (25.21)	0.46 (<0.001)
Convenience	8.54 (3.19)	66.05 (17.07)	0.78 (<0.001)
Overall satisfaction	9.62 (3.03)	68.75 (20.57)	0.62 (<0.001)
Daily activities	7.09 (4)	NA	NA
Medical care	6.66 (1.83)*	NA	NA
Composite score (out of 100, SatMed-Q® only )	74.17 (18.01)	*68.75 (20.57)*	*0.70 (p<0.001)* **

Data are expressed as mean (SD). R: Pearson correlation coefficient (Pearson correlation test).

* (of 8).

** Refers to the correlation between the composite score of the SatMed-Q® and the overall satisfaction score obtained with the TSQM® vII.

NA: not applicable.

### Relationship between Satisfaction with Medication and Adherence

We found a significant correlation between adherence and overall satisfaction with medication whether assessed with the SatMed-Q® (R = 0.23; p = 0.002) or with the TSQM® vII (R = 0.17; p = 0.02). Moreover, there were significant differences in the SatMed-Q® composite score according to the level adherence (Table 4), which was not observed with the TSQM® vII. Multiple linear regression showed that all the dimensions of the SatMed-Q® (except overall satisfaction) were slightly better predictors of patients’ adherence than those of the TSQM® vII (R^2^ = 0.092, P = 0.007; and R^2^ = 0.064, P = 0.01, respectively). Among all dimensions, only “convenience of use” was significantly correlated with adherence in both questionnaires. Moreover, “adverse drug events” were found to be linked to adherence for the SatMed-Q® but not the TSQM® vII (Table 5).

**Table 4:** Relationship between satisfaction with medication and levels of adherence.

		SatMed-Q®		TSQM® vII	
		Mean (SD)	p-value	Mean (SD)	p-value
**Adherence** **(MAQ)**	Good (n = 48)	79.38 (16.82)		72.79 (21.37)	
	Minor problems (n = 109)	73.13 (17.81)	0.016	67.72 (20.03)	0.20
	Poor (n = 15)	65.07 (21.15)*		63.27 (20.94)	

* P<0.017 *vs* “Goodg001 adherence”.

**Table 5:** Relationship between dimensions of satisfaction with medication and adherence.

	SatMed-Q®		TSQM® vII	
	β	p-value	β	p-value
Side effects	**0.16**	**0.03**	0.09	0.23
Effectiveness	−0.08	0.37	−0.08	0.29
Convenience of use	**0.21**	**0.01**	**0.23**	**0.003**
Impact on everyday activities	0.05	0.57	NA	NA
Medical care	0.05	0.46	NA	NA

### Relationship between Satisfaction with Medication and Quality of Life

We found a significant correlation between the two components of quality of life and overall satisfaction with medication with both questionnaires (Table 6). The total SatMed® score significantly correlated with the SF-36® PCS (R = 0.32; p<0.001) and MCS (R = 0.31; p<0.01) dimensions. Similar results were obtained with the TSQM® for PCS (R = 0.30; p<0.001) and MCS (R = 0.31; p<0.01). Multiple linear regression showed that adverse drug events and effectiveness were correlated with MCS and PCS when the SatMed-Q® was used, but with the the TSQM® vII only “adverse drug events” and not effectiveness was correlated with quality of life. In addition, convenience of use was correlated with the MCS (Table 6).

**Table 6:** Relationship between satisfaction with medication and quality of life.

	PCS				MCS			
	SatMed-Q®		TSQM® vII		SatMed-Q®		TSQM® vII	
	β	p-value	β	p-value	β	p-value	β	p-value
Side effects	**0.25**	**0.001**	**0.28**	**<0.001**	**0.18**	**0.01**	**0.22**	**0.003**
Effectiveness	**0.27**	**0.003**	−0.029	0.7	**0.26**	**0.01**	0.08	0.3
Convenience of use	−0.11	0.14	0.13	0.09	0.1	0.2	**0.16**	**0.03**
Impact on everyday activities	0.015	0.88	NA	NA	−0.16	0.09	NA	NA
Medical care	−0.04	0.63	NA	NA	0.08	0.28	NA	NA

### Factors Influencing Satisfaction with Medication

Neither gender, level of education, marital status (living alone or not), type of chronic disease, time since treatment was started, nor medication management, influenced satisfaction with medication. The use of injectable, rather than oral, drugs tended to decrease satisfaction (72.5±19.1 compared to 77.7±15; P = 0.057 with the SatMed-Q®).

## Discussion

The TSQM® and the SatMed-Q® are two generic questionnaires assessing satisfaction with medication, originally developed in English and Spanish, respectively. The validated French translations used in the present study showed high reliability. Both tools share several dimensions (side effects, effectiveness, convenience, overall satisfaction), which were correlated, demonstrating the convergent validity of the French version of the SatMed-Q®. The correlation coefficients are slightly lower than those found in the original validation study by Ruiz *et al* [21], except for ‘effectiveness’, which was clearly lower in our study. This could be explained by the different version of the TSQM® used in both studies.

The original study found a correlation coefficient of 0.74 (p<0.001) between composite scores of the TSQM® and the SatMed-Q® [21]. We could not test this association as the TSQM® version II does not propose a composite score of satisfaction with medication. Therefore, in our analysis we replaced it by the overall satisfaction score. The correlation between the overall satisfaction dimension of the TSQM® vII and the composite score of the StaMed-Q® was 0.64 (p<0.001) in our study. This is sufficient to reasonably conclude that the translated version we used respected the psychometric properties of the original version. Moreover, the relationship between satisfaction and adherence (R = 0.23; p = 0.002) is consistent with the original validation study (R = 0.22; p<0.0001).

The use of both questionnaires to measure patient satisfaction with medication allowed us to highlight differences between these tools. Although in both cases a large part of adherence is not explained by satisfaction (R^2^<0.1), the dimensions of the SatMed-Q® were slightly better predictors of adherence than those of the TSQM®. Moreover, side effects assessed with the SatMed-Q®, but not with the TSQM®, were significantly correlated with adherence. This dimension was shown to be related to adherence in a recent literature review that explored the relation between treatment satisfaction and adherence, compliance, and persistence [6]. These discrepancy between the questionnaires used in our study could be explained by differences in wording when referring to side effects, leading to a possible misunderstanding by patients.

Among other differences, we note that several questions in the SatMed-Q® about the impact of the treatment on everyday activities are irrelevant to most patients, as they relate to physical capabilities and assume that the disease involves functional impairment. Nevertheless, the patients themselves found that the SatMed-Q® was clearer and easier to use than the TSQM® vII, again probably because of differences in wording and in the presentation of the questions. However this result is only valid for the French versions of the questionnaires and deserves further study for versions in other languages.

Another difference between the questionnaires is the influence of “effectiveness” on the quality of life, detected with the SatMed-Q® but not with the TSQM® vII. Conversely, “convenience of use” is a predictor of quality of life for TSQM® vII (although there was only a non significant trend for the physical component), while it is not for SatMed-Q®. The same components of the two questionnaires differed in their ability to predict the level of quality of life of patients, suggesting structural differences between these tools.

Finally, the two additional dimensions of the SatMed-Q® (impact on everyday activities and medical care) did not emerge as factors explaining adherence. This result is somewhat surprising considering that the dimension “medical care” explores the information provided to patients by healthcare professionals, as well as patients’ knowledge about their disease. However, the results of studies measuring the relationship between knowledge and understanding of the management of a disease on one hand, and adherence on another, are very heterogeneous. This dimension could be useful for assessing the quality of care provided by healthcare services and medical teams.

Our study has some limitations. First, it is a single-center study; although the setting was a large university hospital with representative activity, this could attenuate external validity of our results. Another limitation is the imbalance in the number of patients included by disease group. The over-representation of one group of patients could have induced bias to our study. Indeed, we deliberately included patients with chronic diseases having different characteristics, and the predominance of diseases including functional disability (e.g. rheumatoid arthritis) may have influenced the results. However, a disease-adjusted *post hoc* analysis did not change the results (not shown for clarity), suggesting no or only limited influence of the type of disease on our primary outcome.

Another limitation is the questionnaire adapted from Morisky-Green to assess adherence, which only approximately measures the level of adherence. It is exposed to several biases, including memory and willingness to conform socially, and tends to underestimate non-adherence. Nonetheless, a self-reported adherence measure is easy to implement and has proven its validity over refill history or electronic monitoring [5].

In conclusion, this study confirmed the excellent reliability of the two currently available generic questionnaires designed to assess satisfaction with medication. Adherence and satisfaction with medication are positively correlated whatever the questionnaire. We further show that among the different dimensions of satisfaction with medication in a heterogeneous population of patients with various chronic diseases, convenience of use and side effects are prominent predictors of adherence and quality of life, respectively. Therefore, satisfaction with medication assessed with generic tools such as the SatMed-Q® and the TSQM® could be helpful to better identify patients at risk of poor adherence and to personalize patient information and education.
